# Culture and differentiation of rabbit intestinal organoids and organoid-derived cell monolayers

**DOI:** 10.1038/s41598-021-84774-w

**Published:** 2021-03-08

**Authors:** Egi Kardia, Michael Frese, Elena Smertina, Tanja Strive, Xi-Lei Zeng, Mary Estes, Robyn N. Hall

**Affiliations:** 1 Health and Biosecurity, Commonwealth Scientific and Industrial Research Organisation, Canberra, 2601 Australia; 2 Faculty of Science and Technology, University of Canberra, Canberra, 2617 Australia; 3 Centre for Invasive Species Solutions, Canberra, 2617 Australia; 4 Department of Molecular Virology and Microbiology, Baylor College of Medicine, Houston, 77030 USA; 5 Department of Medicine, Baylor College of Medicine, Houston, 77030 USA

**Keywords:** Animal physiology, Biological models, Virus-host interactions

## Abstract

Organoids emulate many aspects of their parental tissue and are therefore used to study pathogen-host interactions and other complex biological processes. Here, we report a robust protocol for the isolation, maintenance and differentiation of rabbit small intestinal organoids and organoid-derived cell monolayers. Our rabbit intestinal spheroid and monolayer cultures grew most efficiently in L-WRN-conditioned medium that contained Wnt, R-spondin and Noggin, and that had been supplemented with ROCK and TGF-β inhibitors. Organoid and monolayer differentiation was initiated by reducing the concentration of the L-WRN-conditioned medium and by adding ROCK and Notch signalling inhibitors. Immunofluorescence staining and RT-qPCR demonstrated that our organoids contained enterocytes, enteroendocrine cells, goblet cells and Paneth cells. Finally, we infected rabbit organoids with *Rabbit calicivirus Australia-1*, an enterotropic lagovirus that—like many other caliciviruses—does not grow in conventional cell culture. Despite testing various conditions for inoculation, we did not detect any evidence of virus replication, suggesting either that our organoids do not contain suitable host cell types or that additional co-factors are required for a productive infection of rabbit organoids with *Rabbit calicivirus Australia-1*.

## Introduction

Two-dimensional (2D) cell culture models suffer many disadvantages because they lack tissue-specific architecture, an extracellular matrix (ECM) and many of the cell–cell interactions that occur in vivo. Organoids are artificial three-dimensional (3D) tissue constructs that can be generated from self-organising stem cells and that allow the establishment of near-physiological conditions in culture^1^. Organoids can be used to model tissue physiology and pathology, study microbial infections, conduct toxicology studies and discover new drugs. In many instances, organoids may replace animal models that are generally more expensive, laborious and have considerable ethical implications^2–4^. Differentiated mature organoids should contain all major cell types of the tissues from which the stem cells were isolated. Organoids can be generated from pluripotent stem cells that are either embryo-derived or isolated from adult stem cells retrieved from tissue biopsies^1, 5^. When supplemented with appropriate growth factors and cultured in an ECM, these stem cells can self-renew and build a 3D tissue-like structure that recapitulates many features of their original tissue. Intestinal organoids, for example, contain many of the differentiated epithelial cells known to be present in adult intestinal tissues, including enterocytes, goblet cells, enteroendocrine cells (EECs) and Paneth cells (Supplementary Fig. S1).

The architecture of the small intestine has been extensively reviewed elsewhere^6, 7^. Supplementary Fig. S1 provides a graphical overview of the tissue organisation and cell types present. It has been shown previously that crypt basal columnar cells (CBCs), multipotent stem cells of the small intestine, undergo self-organisation into symmetrical sphere-like structures ex vivo. Paneth cells then arise and guide the differentiation of other cell types. This leads to the formation of multilobular structures that resemble the crypt-and-villus architecture of the intestine^8, 9^. To grow spheroids (which we call proliferating organoids that still contain a stem cell population) and organoids (which we reserve for terminally differentiated mature organoids), a suitable tissue-specific microenvironment must be created, which can be achieved by using an artificial ECM and a combination of tissue-specific growth factors^10^. In human intestinal organoids, critical signalling pathways depend on Wnt, R-spondin and Noggin (collectively referred to as WRN)^11^, with Wnt being crucially important for maintaining the proliferation of a healthy stem cell pool^12, 13^. Wnt signalling is highly conserved across metazoans, regulating embryonic development and adult tissue homeostasis. The canonical Wnt or Wnt/β-catenin pathway is activated when Wnt proteins bind to proteins of the Frizzled receptor family^14, 15^. This signal can be further enhanced through the binding of R-spondin proteins^15^, which drives the differentiation of stem cells in cell culture^16^. Noggin is also needed to maintain an intestinal stem cell pool^13^. Noggin interferes with the binding of bone morphogenetic proteins (BMPs) to their receptor^17^, thus antagonising the function of cytokines that restrict stem cell proliferation^13, 18^. The removal of Wnt, R-spondin and Noggin allows stem cells to develop from an undifferentiated to a differentiated state, and thereby triggers the formation of organoids from spheroids.

The growth of intestinal organoids has allowed researchers to study enteric viruses that do not infect and/or replicate in conventional cell culture^19^. For example, human caliciviruses (noroviruses), which have resisted cultivation in traditional culture systems for decades, infect and replicate in enterocytes in differentiated human intestinal organoids^20^. It is therefore tempting to speculate that rabbit organoids may support the replication of rabbit caliciviruses. *Rabbit haemorrhagic disease virus* (RHDV) has decimated native rabbit populations in Europe and is used as a biocontrol agent to manage feral rabbits in Australia. RHDV is a hepatotropic virus and it has been hypothesized that it evolved from a benign, enterotropic calicivirus^21, 22^.

Here, we report the development of protocols for the isolation, maintenance and long-term cryogenic storage of rabbit small intestinal spheroids from duodenum, jejunum and ileum, the differentiation of duodenal spheroids to organoids, and the cultivation of organoid-derived cell monolayers. Finally, we tested the ability of an enterotropic rabbit calicivirus, *Rabbit calicivirus Australia-1* (RCV-A1), to replicate in cells of rabbit intestinal organoids.

## Results

### Rabbit intestinal spheroid morphology

**Figure 1:**
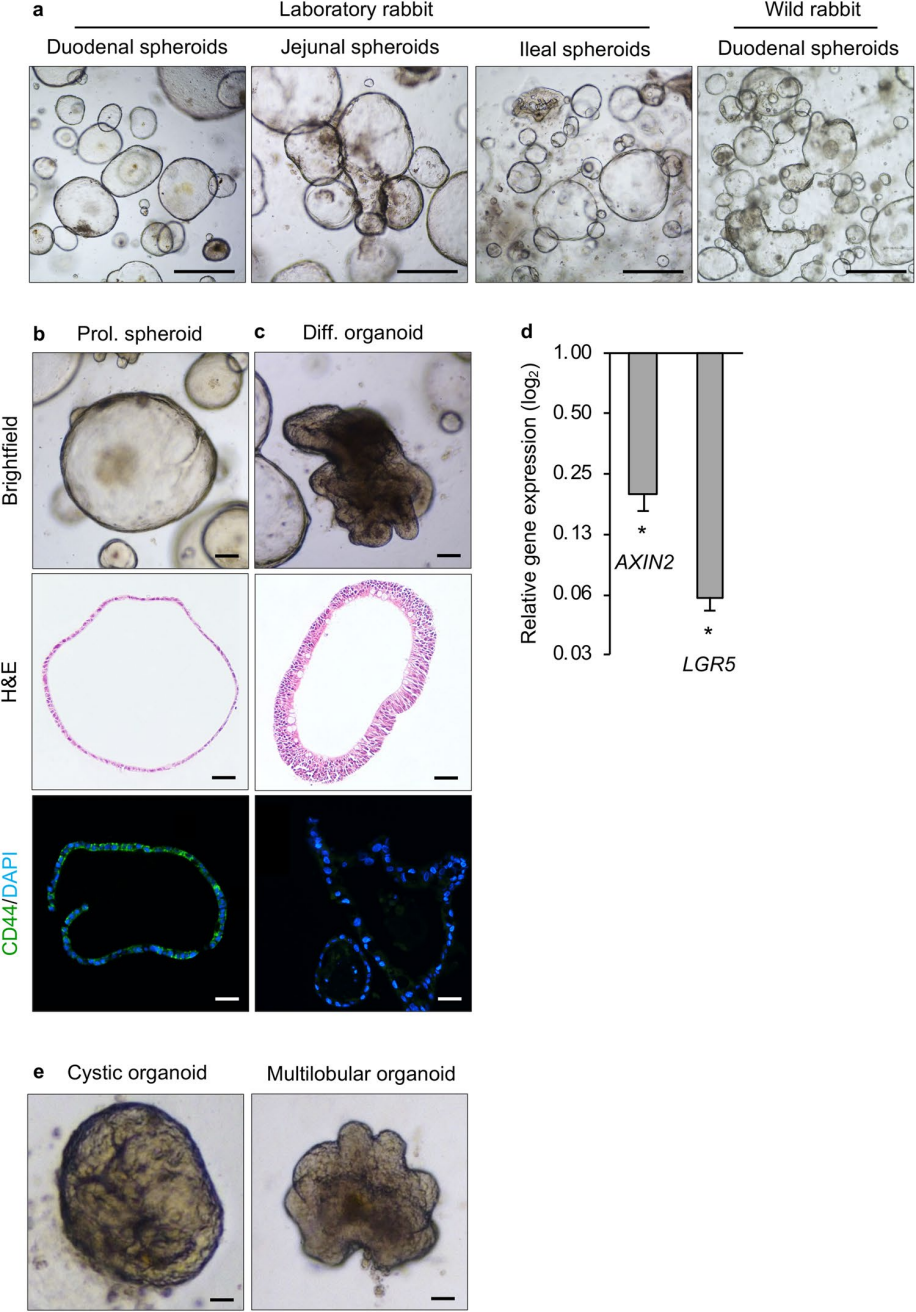
Morphology and characteristics of rabbit small intestinal organoids. (**a**) L-WRN-conditioned medium supported the growth of spheroids from duodenum, jejunum and ileum spheroids from a laboratory rabbit, and duodenum spheroids from a wild rabbit. Scale bars = 500 μm. (**b**) Proliferating rabbit duodenal spheroids and (**c**) organoids were imaged either unstained (brightfield), after staining with hematoxylin and eosin (H&E) or after immunostaining with a CD44-specific (green) antibody; nuclei were counterstained with DAPI (blue). (**d**) Expression of stem cell-related genes (*AXIN2* and *LGR5)* in differentiated organoids. Data are presented as fold change (2^−ΔΔCt^) in gene expression from undifferentiated spheroids, calculated from three individual cell culture wells with three technical RT-qPCR replicates each. Error bars represent standard errors of the mean. Student’s t-test was performed to assess the statistical significance; only statistically significant differences are shown (*p < 0.05). (**e**) Representative images of organoids after four days of culturing in differentiation medium; the images show representative organoids with either a cystic (left panel) or multilobular morphology (right panel). Scale bars = 100 μm. **b**–**e** Used duodenal spheroids/organoids from a single laboratory rabbit.

Intestinal organoid models derived from ‘exotic’ animals such as such as pigs^23, 24^, horses^24, 25^, cats^24^, dogs^24^, cows^24^, sheep^24^, chickens^24^ and ferrets^26^ have been described previously. These were generated to recreate the species-specific molecular and histological tissue phenotypes seen in vivo. Here, we report a protocol for the generation and cultivation of 3D rabbit intestinal spheroids and organoids. Laboratory and wild rabbits were used to isolate small intestinal stem cells. When these cells were cultured in ECM in the presence of WRN factors, spheroids started to form within 3–4 days (Supplementary Fig. S2). We generated spheroids from duodenum, jejunum and ileum tissue samples that were harvested from a laboratory rabbit and duodenal spheroids from two wild rabbits (Fig. 1a).

Our rabbit intestinal spheroids consisted of a thin monolayer of cells surrounding a liquid-filled lumen (Fig. 1b). In addition, these spheroids also contained a highly proliferative CD44-positive stem and/or progenitor cell population (Fig. 1b). Spheroid cultures were differentiated by reducing WRN stem cell growth factors, adding DAPT (a Notch signalling inhibitor) and removing the transforming growth factor β (TGF-β) inhibitor (Supplementary Fig. S2). Four days after initiating the differentiation process, duodenal spheroids grown in differentiation medium developed characteristics that resembled the intestinal morphology, e.g. thickening of the epithelium (Fig. 1c). Furthermore, the CD44-positive stem cell population was no longer detectable in organoids (Fig. 1c). In line with this observation, differentiated rabbit intestinal organoids exhibited a decrease in intestinal stem cell-associated transcripts, namely *AXIN2* (Wnt signalling activity) and *LGR5* (Fig. 1d) relative to the increase of the other cell types. Moreover, we detected protrusions that appeared at the periphery of spheroids, denoting intestinal organoid maturation. In human small intestinal organoids, two distinct morphologies have been reported, cystic and multilobular^27, 28^. Multilobular organoids have one or multiple buds, whereas those without crypt-like protrusions are referred to as cystic. We found both morphologies in our rabbit duodenal organoid cultures (Fig. 1e); an examination of 136 organoids revealed that 85% were cystic and 15% were multilobular.

### Mechanical shearing induces spontaneous differentiation of rabbit duodenal spheroids

While establishing optimal passaging conditions for rabbit duodenal spheroids, we were surprised to find that different methods of cell dissociation led to differences in spheroid morphology. The mechanical shearing of spheroids using hypodermic needles resulted in predominantly multilobular organoids by passage 4; spheroids propagated by mechanical shearing could not be maintained beyond passage 7 (Supplementary Fig. S3). Contrastingly, enzymatic dissociation of spheroids with the TrypLE Express enzyme (Gibco) generated predominantly cystic organoids and spheroids could be sub-cultured at least 17 times. ROCK and TGF-β inhibitors were continuously present after both mechanical and enzymatic dissociation of spheroids. Spontaneously differentiated organoids lost their resident stem cell population, as indicated by a lack of CD44 protein expression and downregulation of *LGR5* gene expression relative to the increase of the other cell types. These differentiated organoids contained goblet cells and enterocytes, as demonstrated through Muc5ac, Periodic acid-Schiff (PAS) and SI staining and gene expression analyses (Supplementary Fig. S3).

### Rho-associated protein kinase (ROCK) and TGF-β inhibitors synergistically support long-term culture of rabbit intestinal spheroids

**Figure 2:**
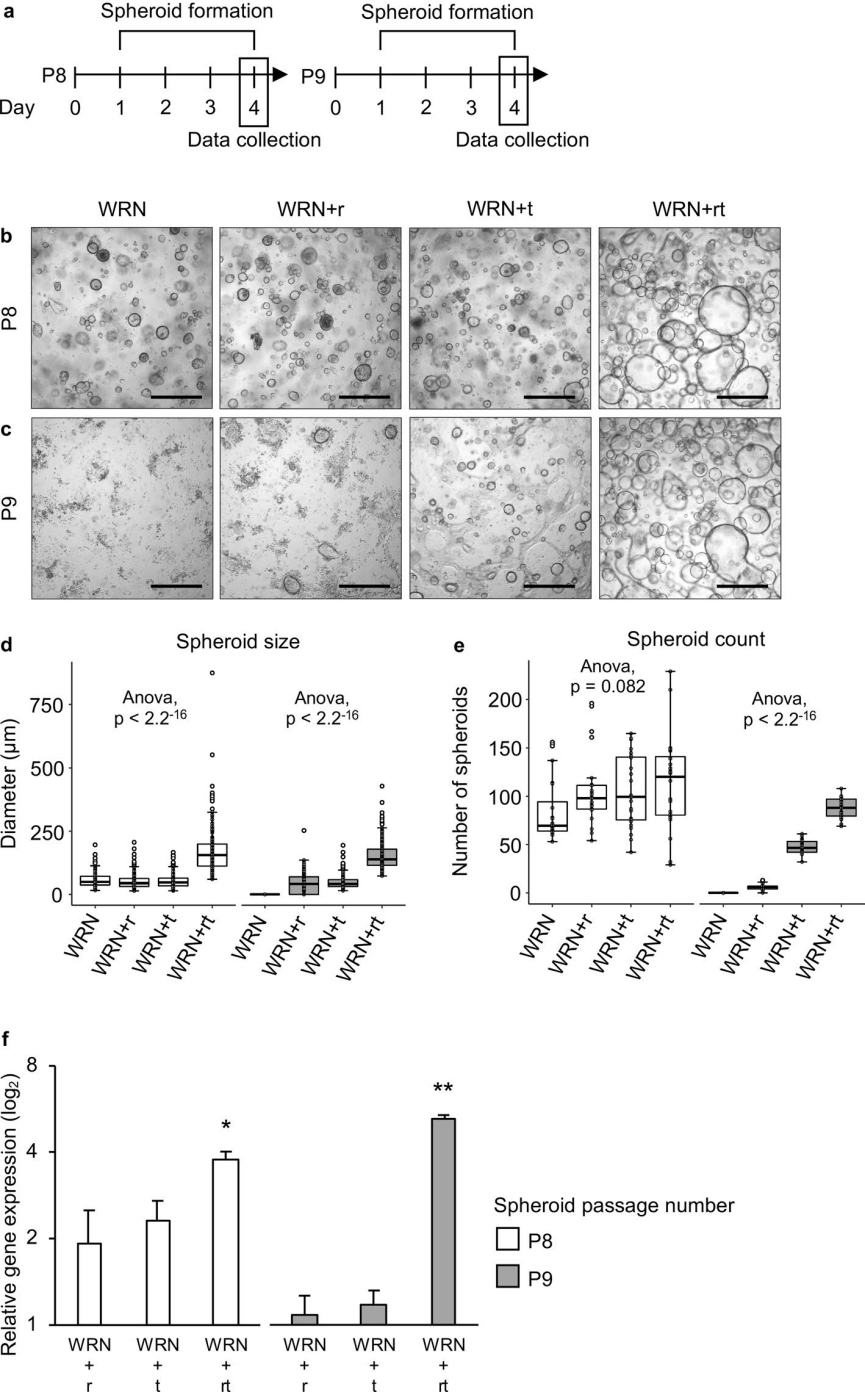
Effect of ROCK and TGB-β inhibitors on the growth of rabbit intestinal spheroids. (**a**) Experimental design. Proliferating spheroids generated from a single laboratory rabbit were passaged 8 and 9 times (P8 and P9, respectively) before they were cultured for four days in proliferation medium without inhibitors (WRN), with ROCK inhibitor (WRN + r), with TGF-β inhibitor (WRN + t), or with both inhibitors (WRN + rt). (**b**,**c**) Representative brightfield images of rabbit duodenal spheroids cultured in different media for two passages. Scale bars = 500 μm. (**d**) Spheroid diameter and (**e**) number of spheroids after culturing in different proliferation media. (**f**) Expression of the proliferation marker *MKI67* in rabbit duodenal spheroids cultured in different media. Data are presented as fold change (2^−ΔΔCt^) in gene expression to spheroids cultured in WRN media without inhibitors, calculated from three individual cell culture wells with three technical RT-qPCR replicates each. Error bars represent standard errors of the mean. One-way ANOVA followed by Tukey’s honest significance test was performed to assess the statistical significance; only statistically significant differences are shown (*p < 0.05, **p < 0.01).

Miyoshi and Stappenbeck demonstrated that, although both ROCK and TGF-β inhibitors are required in early passages of both human and mouse spheroid cultures, these inhibitors were no longer required in later passages^29^. To determine whether rabbit intestinal spheroid cultures behave similarly, we performed proliferation assays in which later passage spheroids (passage 8 and 9) were propagated with and without ROCK and TGF-β inhibitors (Fig. 2a). Spheroids cultured in the continued presence of both ROCK and TGF-β inhibitors consistently grew to a significantly larger size than those cultured under other conditions (Fig. 2b–d). There was no significant difference in the number of spheroids formed under the different media compositions in passage 8 (Fig. 2b,e). However, after subculturing, no spheroid formation was observed in WRN only medium, and significantly fewer spheroids formed in media that contained a single inhibitor when compared to media with both inhibitors (Fig. 2c,e). We also quantified the expression levels of a proliferation marker gene, *MKI67*, and found that gene expression was upregulated when both inhibitors were added to the medium (Fig. 2f). These results suggest that the continuous addition of both inhibitors is required to optimise proliferation of rabbit intestinal spheroids.

### Differentiated rabbit duodenal spheroids show typical hallmarks of differentiated intestinal epithelial lineages

**Figure 3:**
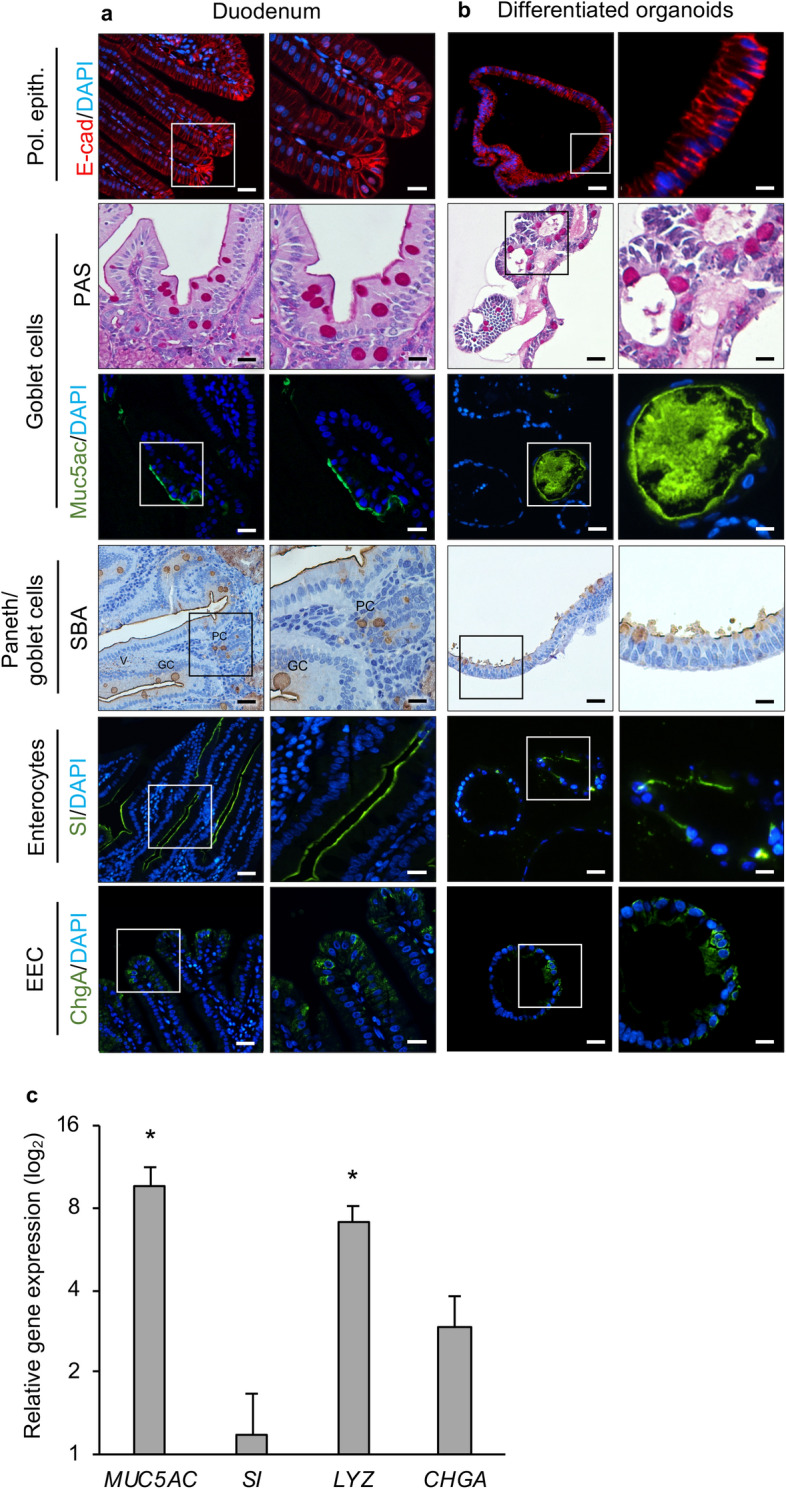
Identification of different cell types in rabbit duodenal tissue and organoids. (**a**) Rabbit duodenal tissue sections and (**b**) rabbit duodenal organoids were immunostained for e-cadherin (red), PAS (magenta), Muc5ac (green), soybean agglutinin (SBA) (brown), sucrase-isomaltase (SI) (green) and chromogranin A (ChgA) (green); nuclei were counterstained with DAPI (blue). Boxed regions are shown at higher magnification in the panels below. Pol. epith., polarised epithelial cells. V, villus. GC, goblet cell. PC, Paneth cell. Scale bars = 100 μm (upper panel) and 200 μm (lower panel). (**c**) Expression of intestinal epithelial maturation-associated genes (*MUC5ac, SI, CHGA* and *LYZ*) in differentiated organoids. Data are presented as fold change (2^−ΔΔCt^) in gene expression from undifferentiated spheroids, calculated from three individual cell culture wells with three technical RT-qPCR replicates each. Error bars represent standard errors of the mean. Student’s t-test was performed to assess the statistical significance; only statistically significant differences are shown (*p < 0.05). All experiments were conducted in duodenal organoids from a single laboratory rabbit.

Human and mouse intestinal organoids contain four major types of specialised intestinal epithelial cells, namely goblet cells, enterocytes, EECs and Paneth cells^11, 27, 30^. To investigate which cell types were present in the rabbit intestinal tissue from which our organoids were derived, we stained rabbit duodenal sections for the expression of marker proteins (Fig. 3a). As expected, tissue sections showed a diffuse staining of e-cadherin along the lateral surfaces of epithelial cells; Periodic acid-Schiff (PAS) and mucin 5ac (Muc5ac) staining in a scattering of goblet cells throughout the villi; soybean agglutinin (SBA) staining of Paneth cell granules^31^ in intestinal crypts and goblet cells throughout the villi; sucrase-isomaltase (SI) staining on the apical brush border of enterocytes; and chromogranin A (ChgA) positive foci, presumably granules in secretory vesicles, in EECs. A similar staining pattern was observed in rabbit duodenal organoids, suggesting the presence of epithelial cells, goblet cells, Paneth cells, enterocytes and EECs (Fig. 3b). Although it was difficult to distinguish between Paneth cells and goblet cells based on SBA staining, some of the SBA-positive cells showed a dark granular staining pattern that we interpreted as characteristic for Paneth cells. We tried to corroborate our findings using lysozyme-specific antibodies, but commercially available antibodies raised against recombinant human lysozyme did not detect rabbit lysozyme in the intestine, a finding that is in agreement with earlier observations^31^. To complement our protein expression analysis, we used RT-qPCR to assess the mRNA expression of genes involved in intestinal maturation and differentiation. As expected, several genes associated with mature intestinal epithelial cells, such as *MUC5ac*, *SI*, *CHGA* and lysozyme (*LYZ*) were upregulated in intestinal organoids compared to spheroids (Fig. 3c).

### Rabbit duodenal spheroid-derived monolayer cultures contain differentiated cells

**Figure 4:**
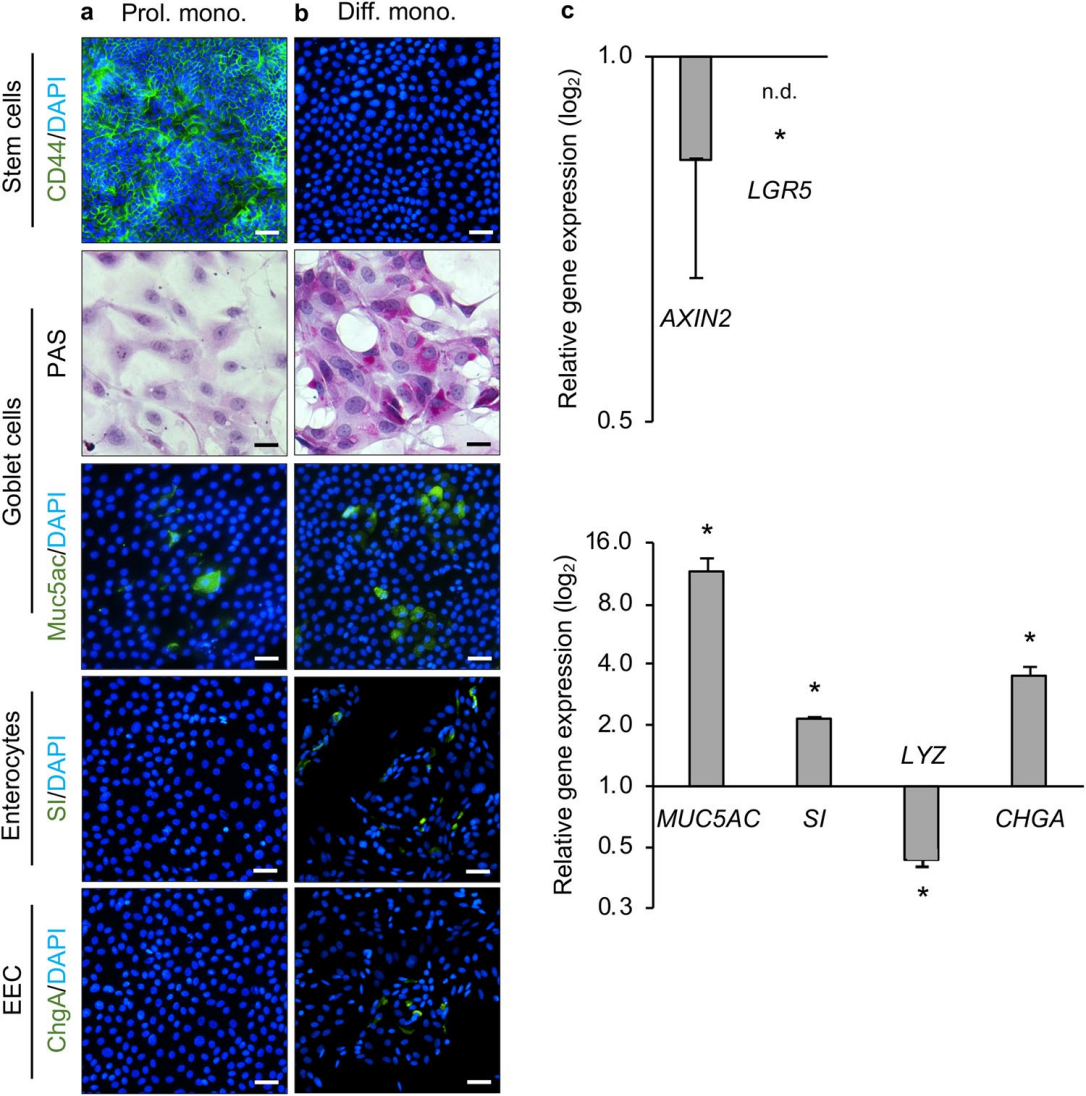
Identification of different cell types in rabbit duodenal monolayer cultures. (**a**) Proliferating (Prol.) and (**b**) differentiated monolayer (Diff. mono.) cultures were immunostained for CD44 (green), PAS (magenta), Muc5ac (green), SI (green) and ChgA (green); nuclei were counterstained with DAPI (blue). Scale bars = 200 μm except for PAS-stained images where scale bar = 50 μm. (**c**) Expression of stem cell-associated (*AXIN2* and *LGR5*) and intestinal epithelial maturation-associated genes (*MUC5ac*, *SI*, *CHGA* and *LYZ*) in differentiated rabbit duodenal monolayer cultures. Data are presented as fold change (2^−ΔΔCt^) in gene expression from proliferating monolayer cultures, calculated from three individual cell culture wells with three technical RT-qPCR replicates each. *LGR5* expression was not detected (n.d.) in differentiated monolayer cultures. Error bars represent standard errors of the mean. Student’s t-test was performed to assess the statistical significance; only statistically significant differences are shown (*p < 0.05). All experiments were conducted in spheroid-derived monolayers from a single laboratory rabbit.

To establish monolayer cultures, rabbit duodenal spheroids were digested with TrypLE Express enzyme and the dissociated cells were plated onto coated culture plates. We found that cells adhered best to surfaces coated with diluted Geltrex LDEV ((lactose dehydrogenase elevating virus)-free reduced growth factor basement membrane matrix; Gibco). Furthermore, we found that a seeding density of approximately 7 × 10^4^ cells/well and 1.4 × 10^5^ cells/well was optimal for rabbit duodenal monolayer cultures in 96-well plates and the Nunc Lab-Tek II 8-well Chamber Slide System, respectively. Immunofluorescence staining demonstrated expression of CD44 in proliferating monolayers, indicating the continued presence of stem cells (Fig. 4a). The occasional goblet cell, but no enterocytes or EECs, were present in the proliferating cultures (Fig. 4a). When these cultures were grown in differentiation medium for four days, we could no longer detect the expression of the stem cell marker CD44 (Fig. 4b), but instead we found evidence for the same differentiated intestinal epithelial cell lineages that were present in organoids (except for Paneth cells). Muc5ac and PAS staining demonstrated the presence of goblet cells, while SI and ChgA indicated the presence of enterocytes and EECs, respectively (Fig. 4b). To corroborate these findings, we analysed the expression of selected genes by RT-qPCR (Fig. 4c). Stem-cell-associated transcripts (*AXIN2* and *LGR5*) were downregulated relative to the increase of the other cell types in differentiated monolayers compared to proliferating monolayers, while transcripts of genes associated with differentiation (*MUC5ac*, *SI* and *CHGA*) were upregulated, suggesting that stem cells differentiated into goblet cells, enterocytes and EECs. However, in contrast to our findings in differentiated 3D organoids, we did not find an increase in the expression of *LYZ* in the monolayer cultures, suggesting that these did not contain Paneth cells (Fig. 4c).

### Rabbit duodenal organoids do not support replication of rabbit calicivirus

To test whether these rabbit duodenal organoid cultures support replication of the benign enterotropic RCV-A1, we inoculated differentiated organoid monolayers and analysed the concentration of viral RNA by RT-qPCR and the expression of the RCV-A1 capsid protein by immunofluorescence. Viral RNA loads at 24 h post-inoculation (hpi) were not significantly different from those at 1 hpi (6 × 10^2^ capsid copies per μl total RNA at 24 hpi versus 2 × 10^4^ capsid copies per μl total RNA at 1 hpi, respectively; p = 0.06 using Student’s t-test). Furthermore, we did not detect accumulation of the capsid protein by immunofluorescence. Although we conducted additional experiments in which we increased the inoculum dose, inoculated cells in the presence of glycochenodeoxycholic acid (necessary for the cultivation of some human norovirus strains^32^), extended the inoculation period from 24 h to 4 days, used whole 3D organoids, infected monolayers grown in transwells to expose the basolateral surface, and scratched monolayers before inoculation to expose tight junctions, we were not able to detect any evidence of viral replication.

## Discussion

Here, we report the generation of rabbit small intestinal spheroids, organoids and monolayer cultures, and our attempts to inoculate these cultures with the enterotropic rabbit calicivirus, RCV-A1. We successfully propagated spheroids from a variety of rabbit small intestinal tissues, including the duodenum, jejunum and ileum. Rabbit large intestinal (caecal) organoids were previously reported by others^33^. We use the terminology ‘spheroids’ to indicate undifferentiated organoids that can be passaged indefinitely, and ‘organoids’ to indicate terminally differentiated mature organoids that are depleted in stem cells and can no longer be passaged^11, 34^.

We were able to culture spheroids for at least 17 passages without any notable changes in morphology or growth rate. We were also able to successfully freeze and revive frozen spheroids using established protocols that were originally developed for human intestinal organoids^34^. Our newly established rabbit organoid cultures reproduce many characteristics of the parental tissue. For example, we detected (1) the presence of multiple differentiated cell types such as enterocytes, EECs, goblet cells and Paneth cells, (2) brush borders on enterocytes, (3) the production of mucin by goblet cells, and (4) the synthesis of lysozyme by Paneth cells. The same cell types, except for Paneth cells, were also detected in differentiated monolayer cultures derived from spheroids. Since high WNT activity is required for Paneth cell differentiation in mouse intestinal organoids^35^, the addition of a GSK-3b inhibitor to the differentiation medium may encourage the emergence of Paneth cells in these monolayer cultures.

We observed that rabbit duodenal organoids exhibited cystic and multilobular morphologies, although most organoids were cystic. Different organoid morphologies have previously been reported for human intestinal organoids^27, 28^ and rabbit caecal organoids^33^, while mouse intestinal organoids are mostly multilobular with protrusions that contain a stem cell niche^30^. The mechanism that drives multilobular organoid morphology remains undetermined, but Paneth cells are suspected to regulate and determine the fate of stem cell differentiation during organogenesis or upon intestinal damage through the secretion of Wnt3^9^. In cell culture, the addition of Wnt growth factor allows stem cells to create their own niche, self-organise and form organoids. Interestingly, it has been reported that a knock down of the murine *WNT3* gene resulted in organoids that had a less pronounced multilobular morphology^9^. It would be interesting to determine if enhanced Wnt signalling, e.g., through higher Wnt concentrations or the use of the homologous (rabbit) cytokine would result in a higher proportion of multilobular rabbit intestinal organoids.

Our observations are generally consistent with previous reports on intestinal organoids of other species, including mice^30^ and pigs^36^. However, we observed some notable differences. For example, rabbit small intestinal organoids spontaneously differentiated when subjected to mechanical shearing. In another study, rabbit caecal organoids cultured in an L-WRN-conditioned media were less proliferative than organoids cultured in media supplemented with pharmacological inhibitors, suggesting that L-WRN-conditioned media is not optimal for maintenance of the stem cell niche in rabbit intestinal organoids^33^. Furthermore, we found that ROCK and TGF-β inhibitors were required for the long-term culture of proliferating rabbit intestinal spheroids. In previous studies, L-WRN-conditioned media supplemented with both ROCK and TGF-β inhibitors was used to generate spheroids from human, mouse, cow, cat and dog intestinal biopsies^24, 29, 37, 38^, but the ROCK and TGF-β inhibitors could be removed in later passages without adversely affecting spheroid proliferation^29^. Therefore, we expected that a similar medium composition would be suitable for the generation of rabbit intestinal spheroids. However, we found that the continuous addition of both inhibitors was necessary for propagating rabbit intestinal spheroids. The ROCK inhibitor that was used in this study, Y-27632, inhibits the two isoforms of the Rho-associated coiled-coil-containing protein kinase ROCK, i.e., ROCK 1 and ROCK 2. Several ROCK substrates are involved in the execution and possibly also in the initiation of apoptosis. Y-27632 has been used to control stress conditions and enhance cell recovery in primary cell isolation and cryopreservation^39^; the inhibitor has also been used to reduce dissociation-induced apoptosis in human embryonic stem cell cultures^39, 40^ and primary primate corneal endothelial cells^41^. However, the response to Y-27632 is cell type-specific and depends on the apoptotic stimulus, which may explain why adding Y-27632 is beneficial in some spheroid cultures, including ours, but not in others.

SB431542 is a selective inhibitor that blocks the activity of the TGF-β type I receptor-like kinases ALK4, ALK5 and ALK7^42^. The BMP/TGF-β signalling pathway is responsible for intestinal epithelial cell differentiation^43^. The addition of a TGF-β antagonist such as SB431542 in the culture medium prevented spontaneous differentiation in mouse embryonic stem cells^44^. Our results show that blocking both ROCK and TGF-β signals is required for the prolonged propagation of rabbit intestinal spheroids. As WRN growth factors promote stem cell proliferation in spheroid cultures, these factors are no longer required for the cultivation of differentiated organoids. Consequently, the removal of these factors leads to stem cell differentiation. Differentiation medium supplemented with DAPT, a Notch signalling inhibitor, stopped stem cells from proliferating and promoted human intestinal organoid differentiation^38,45^. These same conditions were found to facilitate differentiation in our rabbit duodenal organoid cultures.

Intestinal organoids are a useful tool to facilitate functional studies of rabbit pathogens. We investigated whether an enterotropic lagomorph calicivirus, RCV-A1, replicates in our rabbit intestinal organoids. However, despite testing various culture and inoculation conditions, we could not detect any evidence of robust viral replication. We showed that our rabbit organoids contained enterocytes, EECs, goblet cells and Paneth cells. The inability of RCV-A1 to replicate in this system suggests that our organoids lack suitable host cells. Little is known about the cell types that support RCV-A1 replication in vivo. In one study, intestinal samples from infected rabbits were analysed by in situ hybridisation with an RCV-A1-specific antisense probe^46^. Viral RNA detection was restricted to a small number of epithelial cells located at the tips of the villi; however, a further characterisation of these cells was not performed. It is thus possible that RCV-A1 replicates in a rare epithelial cell type that is absent from our organoids. Tuft cells have previously been identified as a host cell for murine norovirus^47^_,_ however, the presence of this cell type in the rabbit intestinal epithelium is still controversial. In our experiments, RCV-A1 did not replicate in enterocytes, suggesting that, unlike human norovirus, RCV-A1 does not productively infect enterocytes, at least not ex vivo. Strain-specific differences were observed for human norovirus infections in intestinal organoids, with GII.3 strains replicating only in the presence of bile acids and ceramide^32^. Future experiments will reveal whether other rabbit calicivirus strains behave differently in our rabbit organoids. Taken together, our findings suggest that enterocytes, EECs, goblet cells and Paneth cells are not target cells of RCV-A1 infection.

Rabbits and other lagomorphs have evolved a digestive system that is radically different to that of other, better researched herbivores^48^. Rabbits are highly susceptible to several gastrointestinal tract diseases, many of which are poorly understood due to the complexity of the digestive tract. For example, epizootic rabbit enteropathy is a disease that predominantly affects rabbits post-weaning and has substantial economic impacts on the rabbit farming industry^49^. However, despite the significance of this condition, the aetiology is multifactorial and poorly understood^49^. Furthermore, a more detailed understanding of the physiology of the rabbit gastrointestinal tract may help to maximise feed conversion efficiency in rabbit production. Rabbit farming is recognised by the Food and Agriculture Organization of the United Nations as having great potential to improve food security and nutrition, particularly in developing countries^50^. The generation of rabbit small intestinal organoids and monolayer cultures will facilitate new investigations into the pathology and physiology of the lagomorph gastrointestinal tract.

## Methods

### Ethics statement

This study was approved by the CSIRO Wildlife and Large Animal Ethics Committee (permit numbers #2016–22 and #2016–02). All animal procedures were carried out at the CSIRO Black Mountain Laboratories according to the Australian Code for the Care and Use of Animals for Scientific Purposes and in compliance with the ARRIVE guidelines.

### Animals

A total of three healthy adult European rabbits (*Oryctolagus cuniculus*) were used for this study: one “New Zealand white” laboratory rabbit (male, 3.6 kg, 1 y 5 m old) and two Australian wild rabbits (one female, 1.9 kg and one male, 1.5 kg; ages unknown). The laboratory rabbit was euthanised by intravenous injection of Lethabarb (Virbac) following intramuscular anaesthesia with 30 mg/kg ketamine (Mavlab) and 5 mg/kg xylazine (Troy Laboratories). Wild rabbits were opportunistically sampled during routine control operations (rabbit shooting) in a nearby national park.

### Isolation and cultivation of intestinal epithelial cells

The isolation of intestinal epithelial cells was performed as described by Miyoshi and Stappenbeck, 2013^29^, with modifications (Supplementary Fig. S4, Supplementary Methods). Dissociated epithelial cells were resuspended in thawed Geltrex. Two 15-μl drops of the matrix-cell suspension were pipetted into wells of a Nunc 24-well-Nunclon Delta-treated plate (Thermo Fisher Scientific) and allowed to solidify for 15 min at 37 °C before 400 μl of proliferation medium (described below) was added to each well. The cultures were incubated at 37 °C and 5% CO_2_ and monitored daily to assess the formation of spheroids. The proliferation medium was changed every 3 days until the matrix dome became crowded with spheroids.

### Passaging and cryopreservation of confluent intestinal spheroid cultures

Spheroid cultures were split and sub-cultured with fresh proliferation medium every week, or sooner if dead cells started to accumulate in the lumen. TrypLE Express Enzyme was used to release the spheroids from the Geltrex dome and dissociate the spheroids into single cells. To freeze dissociated spheroids, cell pellets were resuspended in Recovery Cell Culture Freezing Medium (Gibco). Detailed protocols for isolation and maintenance of rabbit intestinal spheroid cultures is provided in the Supplementary Methods.

### Proliferation medium

A conditioned medium was produced using a mouse fibroblast cell line that was genetically modified to express and secrete Wnt3a, R-spondin 3 and Noggin (abbreviated L-WRN^29^; ATCC CRL-3276). Conditioned media was produced as described previously^29^. The proliferation medium was prepared by diluting the conditioned medium 1:1 with basal medium that contained Advanced DMEM/F12 (Gibco), 20% foetal bovine serum (FBS) (v/v) and 2 mM GlutaMAX Supplement (Gibco). The proliferation medium contained a final concentration of 20% FBS. Spheroids of the small intestine were cultured and expanded in conditioned medium supplemented with 10 μM of ROCK inhibitor (Y-27632; Cayman Chemicals) and 10 μM of TGF-β type I receptor inhibitor (SB-431542; Cayman Chemicals).

### Spheroid proliferation assay

A spheroid proliferation assay was performed to define a media that best supported continuous, long-term spheroid proliferation. Proliferating spheroids at passage 7 were sub-cultured by routine passaging (TrypLE Express enzymatic dissociation). Proliferation medium with or without ROCK and TGF-β inhibitors was added to each well and morphological changes were observed daily. Spheroid cultures were incubated at 37 °C and 5% CO_2_ for 4 days under the different media conditions (passage 8) before being sub-cultured and incubated for a further 4 days (passage 9). To optimise the growth medium for spheroids, we determined spheroid numbers, spheroid size and *MKI67* expression. Briefly, the number and diameter of spheroids were measured using the NIS-Element software (Nikon), and *MKI67* gene expression was evaluated by RT-qPCR (described below). Average spheroid numbers were calculated by counting spheroids in two low-powered fields of view per well, from twelve wells for each medium. Average spheroid diameters were calculated using the measurements from 210 randomly selected spheroids from the 12 wells for each medium.

### Differentiation medium

To promote the maturation and differentiation of duodenal spheroids to organoids, proliferation media was replaced by differentiation media that contained less L-WRN-conditioned medium (i.e., 5%, compared to 50% in proliferation media), which was supplemented with 10 μM of ROCK inhibitor and 50 ng/ml of DAPT (Notch signalling inhibitor). The differentiation medium contained a final concentration of 20% FBS. Although we initially established spheroid cultures from a laboratory rabbit (duodenum, jejunum and ileum) and two wild rabbits (duodenum only), we subsequently focussed our study on the characterisation of duodenal spheroids and organoids from the laboratory rabbit. Duodenal spheroids were incubated in differentiation medium for 4 days to establish differentiated mature organoids.

### Transforming duodenal spheroids to monolayer cultures

Monolayers of spheroid-derived cells were cultured in either flat-bottom, tissue culture-treated 96-well plates or in chamber slides. Prior to use, both plates and chamber slides were coated with Geltrex in phosphate buffered saline (PBS) (1:50 dilution) for at least 2 h at 37 °C. To generate cells for monolayer cultures, proliferating spheroids were grown for 4–7 days and digested using TrypLE Express Enzyme. The resulting crude cell suspension was passed through a 70-μm cell strainer into a 50-ml tube. Approximately 7 × 10^4^ cells in a 100-μl drop of proliferation medium were placed either in the middle of a well of a 96-well plate or in a chamber slide. Differentiation of confluent cells was initiated by changing the proliferation medium to differentiation medium (cells typically reached confluency 1 or 2 days after passaging). Most cells differentiated within 3 days and the number of differentiated cells increased further with time; however, cells started to die 5 days after the switch to differentiation medium.

### Inoculation of rabbit duodenal organoids with rabbit calicivirus

All infection experiments were conducted in triplicate using 100% confluent 4-day-old differentiated duodenal monolayers from the laboratory rabbit, except where otherwise specified. Organoid monolayers grown in 96-well plates or chamber slides were inoculated with 100 μl of a 20% tissue homogenate in PBS that was prepared from the duodenum of an infected rabbit and diluted in differentiation medium with and without FBS to contain a total of 1 × 10^6^ or 1 × 10^7^ capsid gene copies of the RCV-A1 strain MIC1-4^51^ (as quantified by RT-qPCR^52^). In addition to the monolayer cultures, inoculation experiments were performed using 4-day-old differentiated organoids. After a 1-h incubation at 37ºC in 5% CO_2_, the inoculum was removed and was replaced with 100 μl differentiation medium. At 1 and 24 hpi, a 96-well plate was frozen at -80 °C and a chamber slide was fixed for immunofluorescence assays (as described below). Virus loads were quantified by RT-qPCR^52^ and immunofluorescence analysis was performed using the lagovirus (capsid)-specific mouse monoclonal antibody 13C10^53^. In some experiments, we inoculated monolayers in the presence of 500 μM glycochenodeoxycholic acid or extended the post-inoculation incubation period from 24 h to 4 days. Finally, we conducted inoculation experiments in 6.5-mm transwell polycarbonate membrane cell culture inserts (Corning) to expose the basolateral surface to virus, and we scratched the monolayers with a sterile pipette tip to expose tight junctions to virus. Transwell membrane inserts were coated with 100-μg/ml collagen type IV and placed in conventional 24-well plates (Corning) and incubated with 1 × 10^7^ capsid gene copies of RCV-A1 in 600 μl of differentiation medium.

### Histological analysis

At selected time points (day 4–7 for spheroids and day 4 for organoids), spheroids, mature organoids and monolayers were fixed with 4% paraformaldehyde and stained for histological examination (Supplementary Fig. S4). Detailed protocols for histological sectioning and H&E, PAS, immunofluorescence and lectin histochemical staining are provided in the Supplementary Methods. H&E staining was performed to evaluate the structure and composition of spheroids and organoids. PAS staining was performed to identify mucus-producing cells in organoids and differentiated monolayers grown in chamber slides. The following antibodies were used for immunofluorescence staining: anti-E-cadherin (5 μg/ml; LS-Bio), anti-CD44 (10 μg/ml; Thermo Fisher Scientific), anti-mucin5ac (5 μg/ml; Abcam), anti-sucrase-isomaltase (10 μg/ml; Abcam) and anti-chromogranin A (25 μg/ml; Thermo Fisher Scientific); for further antibody details, see Supplementary Table S1. Lectin histochemical staining for Paneth cells and goblet cells was performed using SBA (30 µl/ml) from the Lectin Kit BK 1000 (Vector Laboratories, USA). All staining was examined with an inverted TI-U microscope (Nikon). Image analysis and processing was performed with NIS-Element software (Nikon).

### RT-qPCR

Total RNA was isolated using the RNeasy Mini kit, which included a genomic DNA digestion step with RNase-free DNase. Primer sequence information is provided in Supplementary Table S2. RT-qPCRs were carried out using the SensiFAST SYBR No-ROX kit according to the manufacturer’s instructions on a CFX96 real-time instrument. All gene expression studies were performed with three biological and three technical replicates for each experimental condition (i.e., for characterisation: proliferating spheroids, proliferating monolayers, differentiated organoids and differentiated monolayers; for proliferation assays: spheroids in four different inhibitor treatments). Fold change was calculated using the 2^−ΔΔCt^ method using CFX Maestro software. Transcription of the gene of interest was normalised to expression levels of a housekeeping gene (18S ribosomal RNA).

### Statistical analysis

Statistical analyses and graph production were done using SPSS Statistics version 27 (IBM) and the R packages readxl^54^, ggplot2^55^, dplyr^56^ and ggpubr^57^ in Rstudio running R v3.6.3^58^. Statistical significance for gene expression analyses was assessed using Student’s t-test for two-sample comparisons. For comparisons of multiple independent groups, significance was assessed using one-way analysis of variance (ANOVA) followed by Tukey’s honest significance test. Significance was defined as *p* < 0.05 (*) and *p* < 0.01 (**).

## Supplementary Information

Supplementary Information 1.
