# Supplementary material for: Culture and differentiation of rabbit intestinal organoids and organoid-derived cell monolayers
Source: Sci Rep. 2021 Mar 8;11:5401. doi: 10.1038/s41598-021-84774-w (PMC7940483; doi:10.1038/s41598-021-84774-w)
Supplement: Supplementary file 1 — Supplementary Information 1. [file 41598_2021_84774_MOESM1_ESM.doc]

**Culture and differentiation of rabbit intestinal organoids and organoid-derived cell monolayers**

Egi Kardia, Michael Frese, Elena Smertina, Tanja Strive, Xi-Lei Zeng, Mary Estes, Robyn N. Hall

**Supporting information**

## **Supplementary figures**


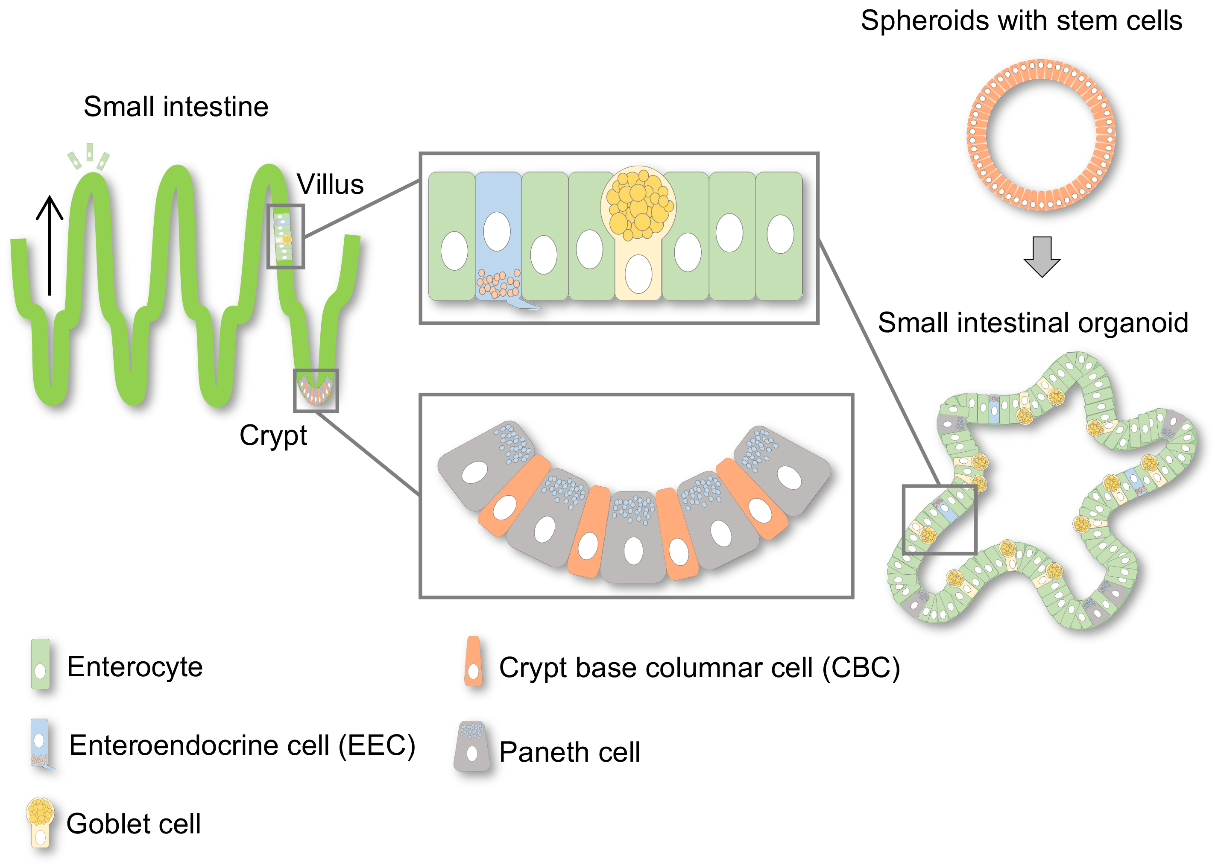


**Supplementary Fig. S1. Epithelial cell types in the small intestine.** The small intestine is lined by a single-layer epithelium with numerous finger-like protrusions (villi) and invaginations (crypts) that greatly enlarge the inner surface area of the small intestine. The villi contain four major types of specialised cells (enterocytes, enteroendocrine cells, goblet cells and Paneth cells), a pool of multipotent stem cells called crypt base columnar cells (CBCs), and rare populations of certain specialised epithelial cells. Enterocytes are the most abundant epithelial cell lineage in the small intestine, where they perform digestive and absorptive functions. The apical membrane of enterocytes is characterised by numerous microvilli that form a brush border, an important host defence against microbes. In addition, the microvilli secrete sucrase-isomaltase, lactase, maltase-glucoamylase and trehalase to aid nutrient absorption^1^. EECs are tall and columnar in appearance with a microvilli-covered apical surface. In contrast to enterocytes, EECs are equipped with a chemosensory extension (neuropod) on their basolateral surface, which connects the cells with the enteric nervous system^2^. In response to ingestion of food, EECs release peptides such as chromogranin A^3^ and/or hormones into the blood stream^4^. Goblet cells are mucin-producing cells with a narrow base and an oval apical portion that contains mucin granules. Mucus secreted from these cells forms a gel-like coating that protects against pathogen invasion^5^. Mucin 2 and Muc5ac are the major components of the mucus in the intestine^6^. Paneth cells reside at the base of the crypts and provide survival signals to adjacent stem cells. Paneth cells also play a role in the innate immune defence; the cells’ secretory granules contain several anti-microbial agents, e.g. α-defensins and lysozyme^7^. CBCs function as the intestinal stem cell population and are usually found at the crypt base intermingled with Paneth cells. CBCs regenerate the small intestinal epithelium through self-renewal and differentiation into specialised epithelial cells. The proliferation of cells near the base of a crypt pushes older cells up towards the tip of the neighbouring villi, a process that continuously replaces senescent cells that undergo apoptosis and are shed from the top of the villi^8,9^. Organoids generated from small intestinal stem cells ideally should contain all major epithelial cell types of the small intestine (i.e., enterocytes, enteroendocrine cells, goblet cells and Paneth cells).


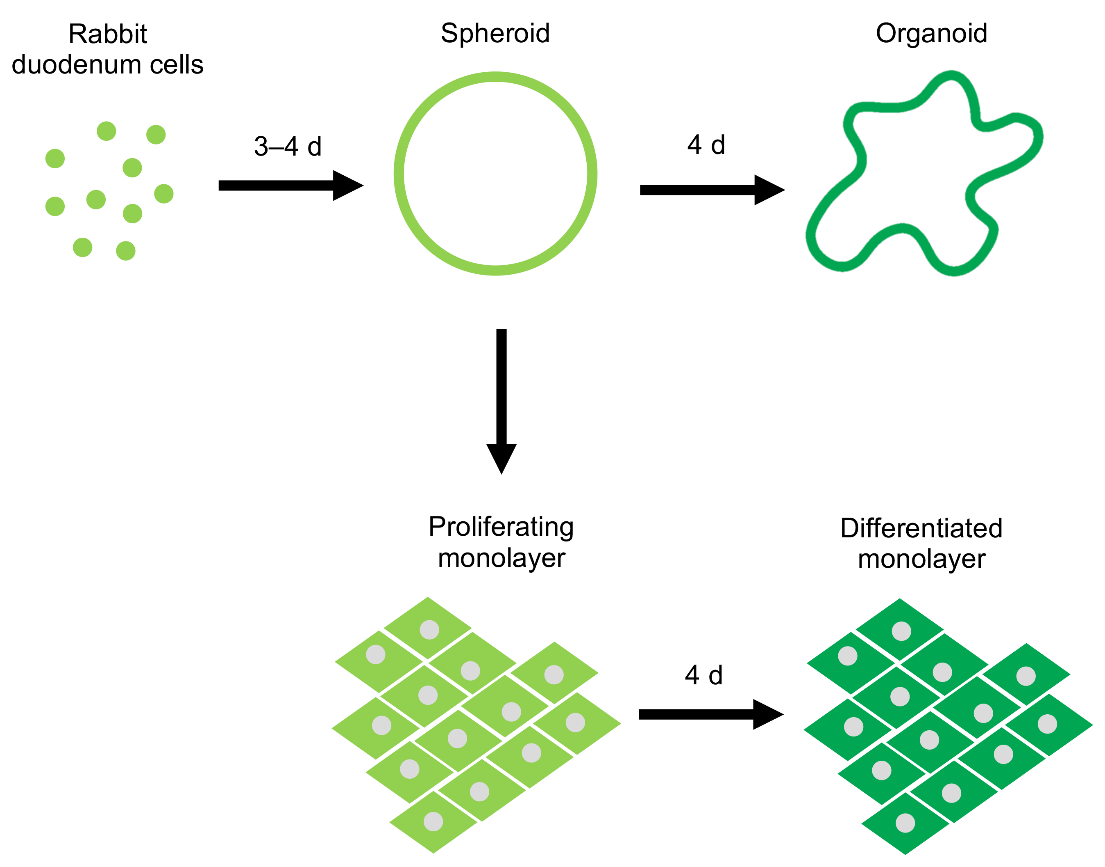


**Supplementary Fig. S2. Time course of rabbit small intestinal organoid and monolayer** **differentiation.** Isolated rabbit duodenum cells were cultured in proliferation medium for 3–4 days to grow spheroids. Mature organoid formation occurred after four days of incubation in differentiation medium. Proliferating monolayer cultures were obtained by dissociating proliferating spheroids; monolayers can be differentiated in the same differentiation medium formulation that was used for spheroid differentiation.


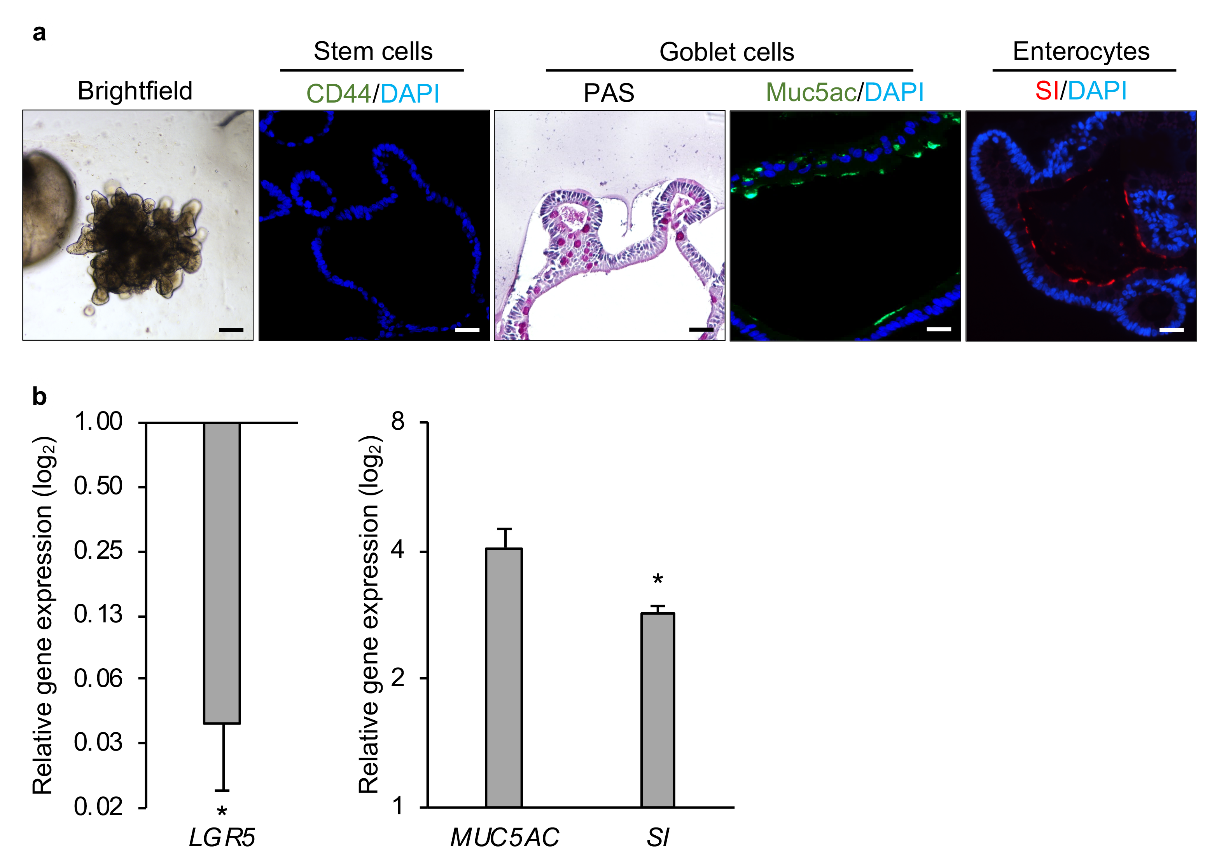


**Supplementary Fig. S3. Spontaneously differentiated organoids resulting from mechanical** **dissociation.** (a) Spontaneously differentiated organoids, either unstained (brightfield) or immuno-stained with CD44 (green), PAS (magenta), Muc5ac (green) or SI (green); nuclei were counterstained with DAPI (blue). Scale bars = 100 μm (immunofluorescence images) and 500 μm (brightfield). (b) Expression of stem cell**-**related (*LGR5*) and intestinal epithelial maturation-associated genes (*MUC5AC* and *SI*) in spontaneously differentiated rabbit duodenal spheroids. Data are presented as fold change (2^-ΔΔCt^) in gene expression from undifferentiated spheroids, calculated from three individual cell culture wells with three technical RT-qPCR replicates each. Error bars represent standard errors of the mean. Student’s t-test was performed to assess the statistical significance; only statistically significant differences are shown (* *p* < 0.05). All experiments were conducted in duodenal organoids from a single laboratory rabbit.


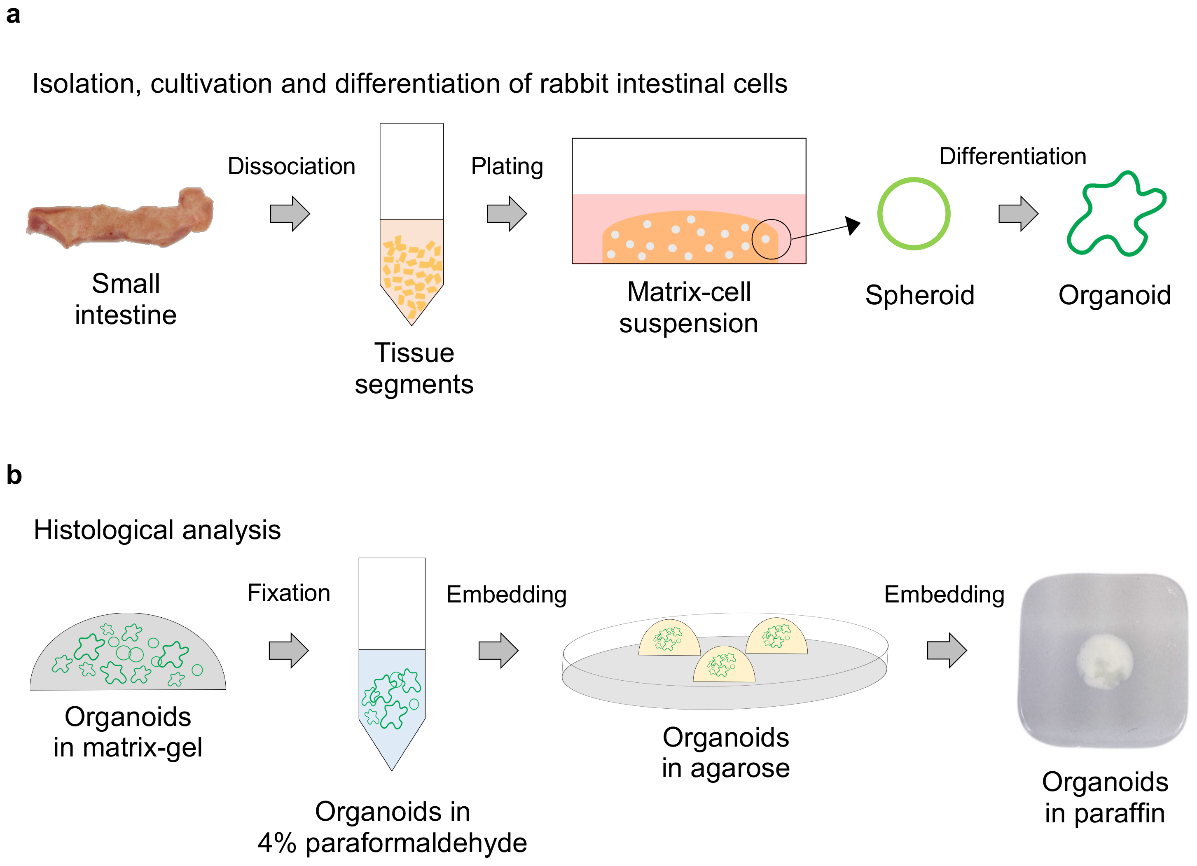


**Supplementary Fig. S4. Generation and histological analysis of rabbit small intestinal organoids.** (a) Sections of the small intestine were cut and incubated in digestion medium overnight. The dissociated intestinal epithelial cells were cultured in extracellular matrix. L-WRN-conditioned medium was added to initiate spheroid formation. Differentiation medium was used to induce the formation of mature intestinal organoids. (b) Organoids grown in matrix gel were fixed in 4% paraformaldehyde for 2 h, embedded in 3% agarose and then paraffin to make blocks for sectioning.

**References for supplementary figures**

1. Feher, J. Digestion and absorption of the macronutrients. in *Quantitative Human Physiology* (2nd ed.) 821–833 (Academic Press, 2017).

2. Gribble, F. M. & Reimann, F. Enteroendocrine cells: Chemosensors in the intestinal epithelium. *Annu. Rev. Physiol.* **78,** 277–299, DOI: <https://doi.org/10.1146/annurev-physiol-021115-105439> (2016).

3. Gunawardene, A. R., Corfe, B. M. & Staton, C. A. Classification and functions of enteroendocrine cells of the lower gastrointestinal tract. *Int. J. Exp. Pathol.* **92,** 219–231, DOI:  [https://doi.org/10.1111/j.1365-2613.2011.00767.x](https://10.1111/j.1365-2613.2011.00767.x) (2011).

4. Latorre, R., Sternini, C., De Giorgio, R. & Greenwood-Van Meerveld, B. Enteroendocrine cells: A review of their role in brain-gut communication. *J. Neurogastroenterol. Motil.* **28,** 620–630, DOI:  [https://doi.org/10.1111/nmo.12754](https://10.1111/nmo.12754) (2016).

5. Specian, R. D. & Oliver, M. G. Functional biology of intestinal goblet cells. *Am. J. Physiol. Cell Physiol.* **260,** C183–C193, DOI:  [https://doi.org/10.1152/ajpcell.1991.260.2.c183](https://10.1152/ajpcell.1991.260.2.c183) (1991).

6. Pelaseyed, T. *et al.* The mucus and mucins of the goblet cells and enterocytes provide the first defense line of the gastrointestinal tract and interact with the immune system. *Immunol. Rev.* **260,** 8–20, DOI:  [https://doi.org/10.1111/imr.12182](https://10.1111/imr.12182) (2014).

7. Clevers, H. C. & Bevins, C. L. Paneth cells: Maestros of the small intestinal crypts. *Annu. Rev. Physiol.* **75,** 289–311, DOI:  [https://doi.org/10.1146/annurev-physiol-030212-183744](https://10.1146/annurev-physiol-030212-183744) (2013).

8. Beumer, J. & Clevers, H. Regulation and plasticity of intestinal stem cells during homeostasis and regeneration. *Development (Cambridge)* **143,** 3639–3649, DOI:  [https://doi.org/10.1242/dev.133132](https://10.1242/dev.133132) (2016).

9. Barker, N., Bartfeld, S. & Clevers, H. Tissue-resident adult stem cell populations of rapidly self-renewing organs. *Cell Stem Cell* **7,** 656–670, DOI: <https://doi.org/10.1016/j.stem.2010.11.016> (2010).

## **Supplementary methods**

### **Isolation and cultivation of intestinal epithelial cells**

The duodenum, jejunum and ileum were collected from a laboratory rabbit and dissected using sterile surgical scissors and tissue forceps. Tissue samples were placed in a 50-ml tube containing ice-cold sterile PBS supplemented with 100 μl/ml antibiotic/antimycotic solution containing 10,000 units/ml of penicillin, 10 mg/ml of streptomycin and 25 μg/ml amphotericin B. The excess fat surrounding the tissue was removed and the intestinal lumen was flushed with ice-cold PBS using a 10-ml syringe with an 18-G blunt needle. The cleaned intestine samples were then opened lengthwise, cut into 1 × 1-cm pieces and incubated overnight in digestion medium containing 1 mg/ml collagenase type I and 100 μl/ml antibiotic/antimycotic solution in Modified Hank's Balanced Salt Solution. Duodenum samples from wild rabbits were processed in the field in a similar manner and transported to the laboratory in a cooler box with ice packs (jejunum and ileum were not collected from wild rabbits). Tissue pieces were digested at 4°C on an orbital shaker-incubator at 200 rpm. After overnight digestion, a cell scraper was used to dislodge the epithelium from the intestine. The epithelial cells were transferred into a 50-ml tube containing 0.25% trypsin/EDTA, incubated for 5 min at 37°C and passed through a 70-μm cell strainer. The digestion was stopped by adding 10% FBS and the cells were pelleted by centrifugation at approximately 250 × g for 5 min at 4°C (Eppendorf 5804 R). After resuspension, red blood cells were removed using ammonium chloride (red blood cell lysing buffer). The cells were pelleted again by centrifugation at approximately 250 × g for 5 min at 4°C. The epithelial cell pellets were then washed twice with PBS, centrifuged at approximately 250 × g for 5 min at 4°C and resuspended in thawed Geltrex LDEV ((lactose dehydrogenase elevating virus)-free reduced growth factor basement membrane matrix). Two 15-μl drops of the matrix-cell suspension were pipetted into wells of a Nunc 24-well-Nunclon Delta-treated plate and allowed to solidify for 15 min at 37°C before 400 μl of proliferation medium was added to each well. The cultures were incubated at 37°C and 5% CO_2_ and monitored daily to assess the formation of spheroids. The proliferation medium was changed every 3 days until the matrix dome became crowded with spheroids.

### **Passaging and cryopreservation of confluent intestinal spheroid cultures**

Spheroid cultures were split and sub-cultured with fresh proliferation medium every week or sooner if dead cells started to accumulate in the lumen. Briefly, the old medium was removed, and the dome was carefully washed using basal medium. To dissolve the matrix and dissociate the spheroids, TrypLE Express Enzyme was added, and the dome was broken up by gentle pipetting until the spheroids were released from the matrix. The spheroids were transferred into a new 15-ml tube and incubated for 10–15 min at 37°C. The digestion was stopped by adding 10% FBS and the cells were pelleted by centrifugation at about 100 × g for 5 min at 4°C. For passaging, the cell pellets were resuspended in thawed Geltrex and the matrix-cell suspension was transferred into a 24-well-plate (two 15-μl drops containing 1 × 10^5^ cells per well). The matrix was solidified for 15 min at 37°C before 400 μl of fresh proliferation medium was added. To freeze dissociated spheroids, cell pellets were resuspended in Recovery Cell Culture Freezing Medium and 1 × 10^6^ cells were transferred to a cryovial. The cryovial was then placed in a freezing container at -80°C overnight and transferred to liquid nitrogen for long-term storage.

### **Histological analysis**

After discarding the medium, the matrix dome containing the organoids was washed with PBS and the matrix was gently broken up by pipetting through 1,000-μl pipette tips that were cut to increase the bore size. The organoids were then transferred to a 15-ml tube, pelleted by centrifugation at approximately 100 × g for 5 min at 4°C, fixed in 4% paraformaldehyde for 2 h at 4°C and embedded in PBS containing 3% low-melting point agarose. The agarose gels containing the fixed organoids were processed in a standard automated tissue histology processor, embedded in paraffin and sectioned into 3-μm thick slices using a semi-automated rotary microtome. The slices were floated in a 37ºC water bath and mounted either on frosted-edge slides for histological staining or on poly-L-lysine-coated slides for immunostaining. The mounted sections were dried overnight at room temperature.

### **Haematoxylin and eosin (H&E) staining**

Samples were deparaffinised for 2 minutes in xylene before hydration through graded ethanol solutions (100%, 90%, 80% and 70%) and tap water (each for 2 min). Next, samples were stained with Harris’ haematoxylin for 5 min, followed by incubation with 0.5% acid alcohol for 2 sec, tap water for 2 min, 0.2% ammonia water for 20 sec and tap water again for 2 min. Samples were counter-stained with eosin for 3 min and dehydrated using graded ethanol solutions (70%, 80%, 90% and 100%; each for 10 sec), followed by a final clearing step with xylene for 2 min, before the slides were mounted using dibutylphthalate polystyrene xylene (DPX).

### **Periodic acid-Schiff (PAS) staining**

Samples were prepared as described above for H&E staining. Samples were stained with 0.5% periodic acid for 5 min, tap water for 1 min, Schiff’s reagent for 15 min and tap water again for 5 min. Samples were counter-stained with Harris’ haematoxylin for 30 sec and dehydrated using graded ethanol solutions (70%, 80%, 90% and 100%; each for 10 sec). Lastly, the samples were cleared using xylene for 2 min and mounted using DPX.

### **Immunofluorescence**

Monolayers grown in chamber slides were washed twice with PBS and incubated in 4% paraformaldehyde in PBS for 15 min at room temperature, then washed again twice with PBS before immunostaining. Spheroid/organoid sections on poly-L-lysine-coated glass slides were heated to 60°C and dipped in xylene for 3 min to remove the surrounding paraffin. The sections were rehydrated with graded ethanol solutions (100%, 90%, 80% and 70%) and PBS (each for 3 min). After paraffin removal and rehydration, both spheroid/organoid sections and monolayers in chamber slides were placed in 10 mM citrate buffer and incubated for 20 min at 95°C to expose antigenic sites. Following antigen retrieval, the slides were cooled and washed with PBS for 5 min. The slides were then permeabilised with 0.25% Triton X-100 in PBS for 10 min at room temperature, washed with PBS for 5 min and blocked with 5% bovine serum albumin (BSA) in PBS with 0.25% Tween 20 for 20 min. The following antibodies were used: anti-E-cadherin (5 μg/ml; LS-Bio), anti-CD44 (10 μg/ml; Thermo Fisher Scientific), anti-mucin 5ac (5 μg/ml; Abcam), anti-sucrase-isomaltase (10 μg/ml; Abcam) and anti-chromogranin A (25 μg/ml; Thermo Fisher Scientific); for further details, see Table S1. All primary antibodies were diluted in PBS containing 0.25% Tween 20 and 1% BSA, and the slides placed into a humidified chamber and incubated overnight at 4°C. Slides were then washed two times with PBS for 5 min before the appropriate secondary antibodies, diluted in PBS with 1% BSA, were added. The slides were incubated at room temperature for another 40 min, washed two times with PBS, counter-stained with 4',6-diamidino-2-phenylindole (DAPI) for 5 min at room temperature and mounted with Fluoroshield.

### **Lectin histochemical staining**

Following deparaffinisation and rehydration, slides were treated with 0.3% hydrogen peroxide in PBS to block endogenous peroxidase activity. Slides were placed in 10 mM citrate buffer and incubated for 20 min at 95°C to expose antigenic sites. After antigen retrieval, the slides were cooled and washed with PBS for 5 min. The slides were then blocked with 1% BSA in PBS. Slides were incubated with the lectin soybean agglutinin (SBA, Table S1) diluted in PBS (30µg/ml). SBA binds specifically to α-D-GalNAc, β-D-galNAc and α and β-Gal. The slides were placed into a humidified chamber and incubated overnight at 4°C. Slides were then washed two times with PBS for 5 min. Streptavidin-biotinylated horseradish peroxidase complex (Sigma-Aldrich) was added for detection and the slides were incubated for 30 min at room temperature. Peroxidase activity was visualised with 3,3′-diaminobenzidine (DAB) (Sigma-Aldrich) after incubation for 3 min. Slides were counter-stained with Harris’ haematoxylin for 1 min and dehydrated using graded ethanol solutions (70%, 80%, 90% and 100%; each for 2 min). Finally, the samples were cleared using xylene for 2 min and mounted using DPX. Negative controls for lectin staining included exposure to horseradish-peroxidase and DAB chromogen without lectin.

### **RT-qPCR**

Spheroids and organoids were harvested using TrypLE Express Enzyme and centrifuged at approximately 250 × g for 5 min at 4°C to form a pellet. Total RNA was isolated using the RNeasy Mini kit that included a genomic DNA digestion step with RNase-free DNase, as per the manufacturer’s instructions. Primers for RT-qPCR were designed to be intron spanning and between 17–21 bases in length using NCBI Primer-BLAST (primer sequence information is provided in Table S2). RT-qPCRs were carried out using the SensiFAST SYBR No-ROX kit according to the manufacturer’s instructions on a CFX96 real-time instrument. The following cycling conditions were used: denaturation at 95ºC for 10 sec min, amplification at 63ºC for 40 sec and extension at 78ºC for 10 sec (40 cycles). ‘No template’ and ‘no reverse transcriptase’ controls were performed with each run. All gene expression studies were performed with three biological and three technical replicates for each experimental condition (i.e., for characterisation: proliferating spheroids, proliferating monolayers, differentiated organoids and differentiated monolayers; for proliferation assays: spheroids in four different inhibitor treatments). Fold change was calculated using the 2^-ΔΔCt^ method using CFX Maestro software. Transcription of the gene of interest was normalised to expression levels of a housekeeping gene (18S ribosomal RNA).

## **Supplementary tables**

| **Antibody name** | **Cell specificity** | **Product number** | **Supplier** | **Conjugated** |
| --- | --- | --- | --- | --- |
| Rabbit polyclonal anti-CDH1/E cadherin | Surface antigen of epithelial tissues, including the GI tract | LS‑C351977 | LS Bio | Unconjugated |
| Rat monoclonal anti-CD44 | Surface antigen of stem cells | MA4400 | Thermo Fisher Scientific | Unconjugated |
| Rabbit polyclonal anti-sucrase-isomaltase | Enterocyte brush border enzyme | ab98872 | Abcam | Unconjugated |
| Mouse monoclonal anti-chromogranin A | Secretory vesicles of endocrine cells | MA5-13096 | Thermo Fisher Scientific | Unconjugated |
| Mouse monoclonal anti-mucin 5ac | Mucus produced in goblet cells | ab212636 | Abcam | Unconjugated |
| Soybean agglutinin | Secretory granules of Paneth cells, mucus produced in goblet cells | BK 1000 | Vector Laboratories | Biotinylated |
| Secondary goat anti-rabbit | Goat polyclonal secondary antibody to rabbit IgG | ab150077 | Abcam | Alexa Fluor 488 |
| Secondary goat anti-rabbit | Goat polyclonal secondary antibody to rabbit IgG | ab150078 | Abcam | Alexa Fluor 555 |
| Secondary goat anti-rat | Goat polyclonal secondary antibody to rat IgG | A-11006 | Thermo Fisher Scientific | Alexa Fluor 488 |
| Secondary goat anti-mouse | Goat polyclonal secondary antibody to mouse IgG | ab150113 | Abcam | Alexa Fluor 488 |
| Streptavidin | Biotinylated compounds | RPN1051V | Sigma-Aldrich | Peroxidase |

**Supplementary table S1.** Antibodies used for intestinal organoid characterisation.

| **Gene name** | **NCBI reference sequence** | **Gene information** | **Sequence (5'→3')** | |
| --- | --- | --- | --- | --- |
| *AXIN2* | XM_017348847.1 | Involved in regulating β-catenin stability in Wnt signalling | F | GGACAGCAGCGTAGATGGAA |
|  |  |  | R | GAGGTAGAGACACTTGGCCG |
| *LGR5* | XM_002711332.3 | Involved in maintaining adult intestinal stem cells | F | TCCAACCTCAGCGTCTTCAC |
|  |  |  | R | CCCGGCAAGACGTAATTCCT |
| *SI* | NM_001082266.1 | Encodes an enterocyte brush border enzyme | F | AAATTCTCGGGGTGACGGAG |
|  |  |  | R | AAGAGAACCTGGTTGGAGGG |
| *CHGA* | XM_008271908.1 | Involved in secretory vesicle production in endocrine cells | F | CGGAAAGGCAAGGGTCGGT |
|  |  |  | R | CTTCTCCATCTTGCTCCAGCG |
| *MUC5ac* | XM_008253829.1 | Involved in mucus production in goblet cells | F | GCGCCTGCACCTACAAC |
|  |  |  | R | GCACTCGGTGCAGTCTGT |
| *LYZ* | XM_002711323.3 | Encodes an anti-microbial enzyme in Paneth cells | F | GCCGCTACTGGTGTAACGAT |
|  |  |  | R | GATCGCTGACGACCCTCTTT |
| *MKI67* | XM_008251084.2 | Expressed in dividing (during G_1_, S, G_2_, and mitosis), but absent in quiescent cells (G_0_) | F | TGGAGTAATCTATGTGGGCCA |
|  |  |  | R | CACCAAGAGCCTTTCACCAAA |
| 18S ribosomal RNA | NR_033238.1 | Housekeeping gene | F | TCGAAGACGATCAGATACCG |
|  |  |  | R | CCCTTCCGTCAATTCCTTTA |

*AXIN2*, axin-related protein 2; *LGR5*, 347 leucine-rich repeat containing G protein-coupled receptor 5; *SI*, sucrase-isomaltase; *CHGA*, chromogranin A; *MUC5ac*, mucin 5A; *LYZ*, lysozyme; *MKI67*, proliferation marker Ki-67. F, forward primer; R, reverse primer.

**Supplementary table S2.** Primer sequences used for rabbit gene expression analyses.
